# Hydrophobia

**Published:** 1836

**Authors:** Otho M. Herron

**Affiliations:** Resident Physician of the Commercial Hospital of Ohio


### Art. IV.—Hydrophobia.

*History of a fatal case of Hydrophobia, with an account of the appearances after death.* By Otho M. Herron, M. D., Resident Physician of the Commercial Hospital of Ohio.

Daniel Wonderly, a laborer, aged 23, was admitted on the evening of February 15, laboring in the second stage of Hydrophobia.

Symptoms etc.—Eyes widely opened, giving him a wild and alarmed expression. Pulse soft, slow, rather small, and 80. Tongue slightly coaked in the centre, margin red. Skin moist. Respiration natural. Great aversion to the sight of water. When urged to drink some, insists upon having it brought up behind him. He then reaches around for the cup and swallows in the utmost haste and terror. The lower jaw is in continued motion, as if to retain its mobile condition, and resist spasm. Fauces dry, and, perhaps, of a little deeper red than natural. Conjunctiva of the right eye injected. Eyes moist. When questioned as to the seat of his distress, he could give it no definite location. Says his head is clear, and no tenderness of the thorax or abdomen could be detected. At the time of his admission he could articulate, but his voice was extremely hoarse. His voice diminished into a whisper, which ultimately subsided into almost perfect aphonia. Insane at intervals, with slight convulsive paroxysms. Tart antimony was given in divided doses, every 15 minutes, until he took gr. viij., but without apparent effect. Chlor. Hyd., gr. xxx., and G. Opii, gr. iij., repeated every two hours. 16.—No visible change. The principle internal medicines used to day, in addition to the above, was extract Belladonnæ, gr. j., every hour; but no narcotic effect was induced. At 4, P. M., a discharge of saliva commenced from the mouth which increased. He made continual, but ineffectual attempts to throw it from him. At between 6 and 7, P. M., his pulse was feeble and hurried, respiration labored, and 40. The disorder of the respiration seemed more like the effect of *fear* than of disease. Urinary evacuation copious. A sour and peculiar effluvium emanates from his body. Eyes of a light green color, and swimming in moisture; opened to the utmost; the conjunctiva of the right one highly injected, and the pupil dilated more than the left. Sees the surrounding objects distinctly enough. Expresses extreme concern for his wife and family. Calls most piteously upon his Maker to relieve him, and entreats those around to kill him out of his torment. His brother-in-law came in at

8 o'clock,—recognised and spoke to him as soon as he entered the room. At this time his countenance was expressive of the most excessive fright and horror. Calls piteously and perpetually upon heaven for relief. Respiration within the last two hours accelerated in frequency, (not counted,) is not more labored than previously but the inspirations are diminished in quantity, and seem as if confined to the superior pulmonary lobes. Spumous discharge from the mouth increased. He died at ten o'clock, this evening. It is impossible to conceive a more frightful picture of anguish than was presented in his case. There were but occasional aberrations of intellect; so, that with the utmost imaginable suffering, there was combined a consciousness almost perfect.

Post. Mortem. (Eighteen hours after death.)

*Countenance* distorted into an agonized expression.

*Head.* The scalp was removed without the loss of a drop of blood. Two ecchymosis about the size of a shilling on the parietal bone of the right side near the junction of the coronal and saggital sutures. Supposed to have been produced by external wound. Dura mater healthy. The longitudinal sinus contained the usual quantity of venous blood. Arachnoid. No perceptible lesion. Pia mater. The vessels upon the surface of the left hemisphere considerably distended. Those on the right less so. Cerebral substance somewhat softened. Upon making a section, the cineritious matter presented an ordinary appearance as to color and depth. The medullary studded with innumerable red points. The ventricles contained a small quantity of reddish serum, perhaps in all 5 or 6 drachms. The choroid plexus of a darker brown and more granular than natural. The vessels of the velum interpositum were very unusually distended. Corpora striata and optic chalami presented no abnormal appearance save the *ramollessiment*, which was common to the whole structure. Pineal gland a shade darker than natural; the color was a pretty good purple. The tubercula qudrigemina, corpora albicantia, petuitary gland and pons varolii were carefully examined, but exhibited no diseased aspect differing from that of the cerebrum in general. The cerebellum less consistent than the cerebrum. I believe this is invariably the case in fresh subjects. The cineritious substance of a brighter red than that of the cerebrum.

The medulla spinalis was cut off as low down as it could be reached with the knife, and examined with all possible care, but no morbid appearance could be seen. Thorax. Both lungs exceedingly engorged, so that there was scarcely a perceptible collapse when the sturnum and costal cartilages were removed. No adhesion. No effusion into the cavity of the pleura. Emphysematous vesicles in several places upon the exterior surface, but most numerous in the interstices of the lobes. A small effusion of lymph at the lower border of the inferior lobe of the right lung. Effused a reddish serosity when incised. Heart sound. Large arteries sound.—No escape of air from them.

Trachea and bronchia. The lining membrane at the upper part of the trachea was pale and tumified. The pouches of morgagni filled up, and the parts in the immediate region so swollen as to destroy the possibility of healthy function in this part, and to account clearly for the difficulty in articulating. At about two-thirds of the way down the trachea, the membrane abruptly assumed a dark brown color, and was covered with a layer of tenaceous mucous, colorless on the surface, but having beneath, a reddish lymphatic secretion, which gave the membrane its peculiar hue. This adhered firmly to the membrane, which was easily abraded by a scrape of the scalpel. This disorganization was traced into the most minute ramifications of the bronchia that could be examined.

Pharynx. At the upper part, the mucous membrane arid, and extremely pale. Towards the middle, the caliber exceedingly contracted—the handle of a small scalpel was passed down with difficulty. At this part, the membrane is traversed by numerous longitudinal corrugations, and incrusted by a stratum of doughy albuminoid matter of such a consistence as to be rolled into little cylinders between the fingers. At the portion lying contiguous to the larynx, this crust had a dark discoloration. Beneath this part, which embraces, perhaps, three inches, the pharynx gradually assumes its natural aspect.

Stomach. About a gill of green and foetid fluid in this viscus. A number of round excavations were scattered over the surface of the mucous membrane, from the size of a pin head to that of a large pea; most numerous towards the pyloric extremity of the greater curvature, and not having an appearance of recent ulceration.

Liver, Spleen, Kidneys and small intestines healthy. Colon and Rectum have the well marked traces of an old diarrhœa. Bladder healthy and empty.

The par vagum was carefully and minutely examined, and presented no diseased aspect either in the neurilemma or fibre.

*Glands.* Sublingual, Parotid, and submaxillary, exhibited no trace, whatever, of morbid change.

Considerable pains were taken to obtain an accurate history of the case up to the time of his admission. The following are the facts that may be relied upon, as related by Mr. Brown, the employer of the patient, and several of his relatives, all of whom live between three and four miles from the city. On the 22d January, the patient was bitten near the metacarpal joint of the little finger, by a small female lap-dog. It was so unusual an occurrence, as to excite suspicion. She was watched and observed to bite a puppy not long after. The puppy was confined, went mad in nine days, and died.—No mad dog had been heard of in the vicinity for years, besides this one. He applied, soon after his bite, to a *steam doctor;* next tried the *mad stone;* next posted off to an *Indian doctor,* living at a considerable distance, and finally sent for a *regular doctor.* After subjecting himself to the multifarious and diverse plans of treatment prescribed successively by his doctors he believed he was safe, and went to his regular employment. Up to this time, he felt no symptoms that alarmed him except a *vertigo,* which visited him almost daily, until a short time previous to the attack. He worked for a few days; but on the 21st he felt a *dull pain*, in the wound, at the point where he was bitten. This gradually ascended the arm, communicating a sensation of dull heavy uneasiness, entered the body, and was then felt in like manner through the left side, and finally throughout the body. He now, (the morning of the 22d day,) first manifested the pathognomonic symptom—abhorrence of water. He came to the Hospital four days after the invasion of the second stage of the disease.

The true nature of the hydrophobic virus has long been a subject of interest and speculation. The experiments of Magendie and Breschet demonstrate the fact, that the peculiar poison which gives rise to this disease is contained in the salivary secretion which is so abundantly discharged from the mouth of the patient. They inoculated two healthy dogs with the saliva from the mouth of a dying patient, one of which became rabid in 44 days, and the other in 75. The specific virus of rabies is mixed in some manner with the saliva, but no analysis has, as yet, been equivalent to its demonstration. In a practical point of view, its discovery is infinitely less desirable than that of its antidote. Our microscopic school of philosphers would doubtless regard it as unequivocally *animalcular*, as they do the proximate cause of variola, yellow fever, and all other diseases. Why is it that the hydrophobic virus, in like manner with that of most other diseases, may pass, harmless, through all other parts of the animal structure, and exert its terrible morbific agency upon particular organs?—it is indubitably certain that there exists a correspondence between certain poisonous substances that pervade the kingdoms of nature in various forms, and particular organs and functions of the animal system; and that in this correspondence lies the deep secret of the *specfiic* agency of the morbific as well as curative substances; and hence, the Homœopathic system is pregnant with at least one truth that is of the utmost importance in medicine. This correspondence of the active ingredients of nature with the various structures and functions of the human system presents an arena for the research and labor of medical *savans* which is beginning to be traversed with hopeful assiduity.

The fear of water, and, at the same time, the desire to drink *may be physically* and pathologically accounted for by the morbid appearances on dissection. We have a portion of the pharynx exceedingly dry, and, at the same time, so contracted and disorganized as to prevent deglutition. Death, in the preceding case, resulted from the pulmonary congestion, attended by the diseased condition of the lining membrane of the air passages.

How far the disorganization of the stomach was connected with the original disease is extremely uncertain.

If the inflammation of the pharynx was a consequence of its contiguity to the larynx, why were not other contiguous parts involved?

Is it highly probable, that the brain suffers in this disease only symptomatically?

